# Rehabilitation approaches for children living with HIV in sub-Saharan Africa: a protocol for scoping review

**DOI:** 10.1186/s13643-019-1219-x

**Published:** 2019-12-23

**Authors:** Stacy Maddocks, Saul Cobbing, Jill Hanass-Hancock, Verusia Chetty

**Affiliations:** 1 Discipline of Physiotherapy, School of Health Sciences, University of KwaZulu-Natal, Westville Campus, Private Bag X54001, Durban, 4000 South Africa; 2 South African Medical Research Council, 123 Jan Hofmeyer Rd, Westville, KwaZulu-Natal South Africa

**Keywords:** HIV, Rehabilitation, Paediatric, Sub-Saharan Africa

## Abstract

**Background:**

A large number of children living with chronic conditions such as HIV experience impairments and disabilities. Current sub-Saharan African healthcare systems are challenged with paediatric care that does not integrate rehabilitation into management of chronic diseases such as HIV. Furthermore, little attention is paid to societal inclusion, community engagement and educational needs of these children. Integration of paediatric care and rehabilitation in a holistic approach can help to overcome the challenges associated with living disabilities. This scoping review proposes a synthesis of existing evidence on rehabilitation intervention strategies to increase functioning and to address disability-related barriers in children living with HIV and disability in sub-Saharan Africa.

**Methods:**

A scoping review will be conducted to systematically map evidence on rehabilitation intervention for children living with HIV in sub-Saharan Africa. Studies in sub-Saharan Africa from December 2012 to 2019 on rehabilitation interventions for children aged 5 to 10 years living with HIV will be included in the review. Peer-reviewed primary studies, as well as grey literature, will be identified from electronic databases including Google Scholar; PubMed; Medline; CINAHL and Cochrane. The search strings using keywords such as “HIV”, “impairment”, “disability”, “neurocognitive impairment”, “behavioural”, “rehabilitation” and “intervention” will be conducted using Boolean logic. Two groups of independent reviewers will conduct all title, abstract and full article screening. The study selection process will be mapped using Preferred Reporting Items for Systematic Reviews and Meta-Analyses extension for Scoping Reviews. A predesigned data-charting table will supplement the extraction of data. NVIVO software will aide in the thematic analysis of the data.

**Discussion:**

The information from studies will be discussed in relation to the research questions using a critical narrative to explore the emergent themes. The quality of studies will be appraised using the mixed method appraisal tool. The scoping review will provide a baseline of evidence on rehabilitation interventions for children living with HIV in sub-Saharan Africa. The scoping review will inform healthcare providers, scholars and policy developers about the current use of rehabilitation interventions and what gaps need to be addressed with further research and intervention development.

**Systematic review registration:**

OSF Center for Open Science: https://osf.io/ed7zb/

## Background

Children living with chronic conditions such as human immunodeficiency virus (HIV) are faced with challenges that influence their well-being, day-to-day community life and societal participation. In 2017, there were approximately 36.9 million people living with HIV, with about 1.8 million being children under the age of 15 years [1]. The response to the HIV epidemic has been one of the greatest public health achievements in the 21st century in Africa. It is also one of the greatest public health concerns in Africa and therefore lends itself to further investigation into how the management of this now-chronic disease is linked to rehabilitation interventions. The surge of campaigns for the prevention of mother-to-child transmission, and the advent of antiretroviral therapy (ART), in resource-poor settings has resulted in huge progress in the medical management of HIV paediatric care. The approach has seen the decrease of HIV infections amongst children around the world since 2010. However, the predicted decline in new infections in children is still not ideal [2]. Children infected with the virus who are on ART live longer, but may experience the effects of HIV as a chronic disease. Chronicity associated with HIV in children influences their day-to-day lives, including social interaction in the home, community participation such as sport and play, and school readiness, which influences not only educational but also social development [3–5].

Children living with HIV are susceptible to comorbidities associated with the virus and side effects related to the treatment, as well as environmental factors such as a lack of family support, stigma and poverty [3–5]. These comorbidities, treatment side effects and contextual factors are often experienced as either episodic or long-term functional limitations or disabilitie s[3, 6]. Episodic experiences of disability include periods of exacerbation and amelioration. These disabling effects of living with chronic HIV need to be addressed through a holistic approach to HIV care. Studies in sub-Saharan Africa suggest that there are many children living with HIV, including those on ARTs, who are experiencing various disabilities manifesting as developmental delays, and neurocognitive and behavioural impairments, as well as communication and sensory disorders [3, 6, 7]. Impairments and disabilities experienced by children living with HIV can be addressed through a diverse set of rehabilitation interventions. A scoping review conducted in 2012 focused on children living with HIV and rehabilitation globally and revealed a lack of rehabilitation services and appropriate research aimed at children living in sub-Saharan Africa, despite the need for rehabilitative care to be integrated into healthcare systems [8]. Since then HIV-testing and ART has been upscaled in sub-Saharan Africa, which has changed not only the number of people surviving with HIV but also increased the need for chronic care interventions.

Despite the need for rehabilitation, it is not routinely integrated into HIV-care, including paediatric HIV healthcare approaches. Furthermore, there is a lack of integration of rehabilitation into HIV policies and strategic plans, as well as limited financial and human resources. Additionally, there is a lack of knowledge and understanding by healthcare professionals and caregivers on how to identify and address HIV-related disabilities [9, 10]. Without rehabilitation interventions for children living with HIV who develop functional limitations, disabilities will develop and consequently influence school readiness, a key predictor of school performance, and employment and standards of living in adulthood [11]. An integral shift is needed to offer children living with HIV healthcare that incorporates a comprehensive continuum of integrated care including rehabilitation, especially in the sub-Saharan African region where HIV is still a public health concern [7, 8]. This study is part of a larger study that aims to assess the feasibility (acceptability, practicality, preliminary efficacy) of an integrated model of rehabilitation and paediatric HIV-care in order to improve the diagnosis of, and interventions for, disability in children living with HIV between the ages of 5 and 10 years [7].

The objective of this systematic scoping review is to identify and map the new reported rehabilitation interventions for children living with HIV who have disabilities in sub-Saharan Africa (between December 2012 and 2019). The study will offer evidence for healthcare professionals, policy makers and scholars that will aid rehabilitative care and its further integration into healthcare systems.

## Methodology

The study design is a systematic scoping review of an array of literature sources, which will include all levels of evidence, excluding opinion papers, review papers and commentaries. Peer reviewed published literature in the last 7 years (December 2012 to 2019) and grey literature on interventions for rehabilitation in sub-Saharan Africa for children living with HIV aged 5 to 10 years will be sourced. Arksey and O’Malley’s [12] scoping review framework will guide this review. The broad research question, identifying relevant studies, study selection, charting the data, as well as collating and reporting of the results, will be adhered to in the study design and review [12, 13].

### Identifying the research question

The research question underpinning the review is “What rehabilitation interventions have been integrated into chronic HIV-paediatric care for children living with HIV who have disabilities?”

Sub-questions that will guide the review are as follows.

1. What evidence exists on the rehabilitation approaches to address physical impairments in children living with HIV in sub-Saharan Africa?
2. What evidence exists on the rehabilitation approaches for neurocognitive impairments in children living with HIV in sub-Saharan Africa?
3. What evidence exists on rehabilitation intervention for behavioural challenges experienced by children living with HIV in sub-Saharan Africa?
4. What evidence exists on rehabilitation intervention for communication impairments experienced by children living with HIV in sub-Saharan Africa?
5. What evidence exists on rehabilitation intervention for sensory impairments experienced by children living with HIV in sub-Saharan Africa?

### Eligibility of research question

The study will incorporate the Participants Concept Context (PCC) model [13]. The participants will be children living with HIV aged 5 to 10 years experiencing impairments and disabilities. These impairments will encompass physical manifestations and neurocognitive manifestations, as well as, or including, behavioural challenges, communication and sensory impairments.

The concept is rehabilitation approaches and intervention strategies used to address impairments (physical, neurocognitive and behavioural, as well as communication and sensory) and disabilities in children living with HIV, aged 5 to 10 years.

The context is all healthcare systems, encompassing primary (community outreach and clinic), secondary and tertiary systems in sub-Saharan Africa. Studies set in the home will also be included in the review as a recent generalised literature search revealed home-based interventions for children living with HIV in sub-Saharan Africa.

### Inclusion criteria

• Articles published between December 2012 and 2019

• Studies reporting evidence on rehabilitation intervention strategies for physical, neurocognitive, behavioural or communication and sensory impairments or disabilities in children living with HIV aged 5 to 10 years in sub-Saharan Africa

### Exclusion criteria

• Opinion pieces on rehabilitation approaches for children living with HIV aged 5 to 10 years in sub-Saharan Africa

• Commentaries on rehabilitation approaches for children living with HIV aged 5 to 10 years in sub-Saharan Africa

• Review papers on rehabilitation approaches for children living with HIV aged 5 to 10 in sub-Saharan Africa

### Identifying relevant literature

This study will include primary research articles published in peer reviewed journals, as well as grey literature. The following electronic databases will be used: Medline, CINAHL, PubMed and Cochrane. The search terms will include “children”; “paediatric”; “HIV”; “impairment”; “disability”; “neurocognitive impairment”; “behavioural”; “rehabilitation” and “intervention”. Boolean terms “AND”; “OR”; “NOT” will be used to separate keywords. A pilot search using the above keywords to determine the feasibility of the study was conducted (Table 1). The search strategy will be adjusted to each database. Details such as the keywords, date of search, search engine and the number of publications retrieved will be documented.

**Table 1:** Results of a pilot search

Date	Database	Keywords search strategy	Search results
29/08/2019	PubMed	1. "children"[All Fields] OR "child"[MeSH Terms] OR	
		2. "paediatric"[All Fields] AND "HIV"[All Fields] OR	
		3. "hiv"[MeSH Terms] AND "human immunodeficiency virus"[All Fields] AND "impairment"[All Fields] OR "disability"[All Fields] OR	
		4. "neurocognitive impairment"[All Fields] OR "behavioural"[All Fields] AND	
		5. "rehabilitation"[All Fields] OR "Rehabilitation"[MeSH Terms] OR	
		6. "intervention"[All Fields] AND	
		7. (("2012/01/01"[PDAT]: "2019/12/31"[PDAT]) AND	
		8. "humans"[MeSH Terms])	
		9. Add 1,2,3,4,5,6,7, and 8	

### Charting of the data

A data-charting template (Table 2) [13] will be used to extract and capture information from studies through each phase of the review. The chart will be continuous and updated regularly through the scoping process in order to capture all possible results for the research question.

**Table 2:** Data charting [13]

Author(s)	
Year of publication	
Origin/country of origin (where the study was published or conducted)	
Aims/purpose	
Study population and sample size (if applicable)	
Methodology/methods	
Intervention type, comparator and details of these (e.g. duration of the intervention) (if applicable)	
Duration of the intervention (if applicable)	
Outcomes and details of these (e.g. how measured) (if applicable)	
Key findings that relate to the scoping review question/s	

### Collating, summarising and reporting results

The data extracted from the studies will be guided by the research questions and assimilated into the sub-questions: evidence that exists on the rehabilitation approaches for each impairment type; the study methods (data collection and sampling tools); how the interventions addressed functionality; how the interventions addressed the social and environmental context of the children; and how the interventions were integrated with HIV-paediatric care. The data will be presented in the final write-up using the themes in Table 3 and the above sub-questions, with flexibility in the capturing of data as the scoping review may yield concepts presently unknown. The data will draw from Table 1 and be further interpreted into a narrative discussing the content of the studies.

**Table 3:**
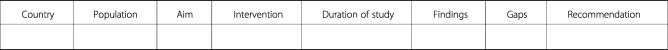
Data reporting on rehabilitation interventions for children living with HIV

### Quality appraisal

The Mixed Methods Appraisal Tool (MMAT) (Appendix) version 2018 will be utilised to appraise the quality of studies from the search strategy described above. MMAT is suitable as the study exclusion criteria include non-empirical papers such as commentaries and opinion papers [14]. The process requires critical appraisal, and therefore three reviewers (research team SM, VC, SC) will be involved in the appraisal process. One of the reviewers has experience in MMAT application, and this will add rigour to the appraisal. The MMAT ‘category one’ allows for the inclusion of qualitative studies; ‘category two’ allows for quantitative randomised controlled trials; ‘category three’ allows for quantitative non-randomised trials; ‘category four’ allows quantitative descriptive studies and ‘category five’ mixed methods studies [14]. Methodological quality criteria will be captured as per the MMAT by two reviewers, with the third overseeing the process. The MMAT will be used to capture a detailed representation of each criterion to inform the appropriateness of the study, the methodological rigour and apt inferences [14].

## Discussion

Children living with HIV who have disabilities are at risk of being excluded from participation in communities and the societies in which they live [4, 7]. Furthermore, their school readiness is at risk, which will be a challenge throughout the lives of these children. Only a little evidence is available about how to address these impairments and disabilities in order to prepare them for school and day-to-day life [7, 11] and the existing evidence needs, therefore, to be systematised to inform further research and programming. This scoping review protocol endeavors to lay down a foundation for mapping evidence in order to promote an integrated rehabilitative approach to care and provide a foundation for further investigation into children living with HIV experiencing impairments and/or disabilities.

Evidence from 2012 to 2019 will be sourced and mapped in order to understand the strategies used in the sub-Saharan context to address the rehabilitation needs of children living with HIV aged 5 to 10 years. This will also inform intervention development for children living with chronic conditions, such as HIV, who need to achieve school readiness.

The follow-up scoping review will also allow health professionals involved in the rehabilitation of children living with HIV to have access to an integrated study on evidence of intervention strategies. The review will also provide scholars with a base to identify the future research needs of children living with HIV and needing rehabilitation. The scoping review could also provide data for policy makers on the needs to be addressed in healthcare systems for children living with HIV experiencing disabilities.

## Appendix

**Table 4:** On initial search

Keywords terms	Date of search	Search engine used	No. of publications
((((("child"[MeSH Terms] OR "child"[All Fields] OR "children"[All Fields]) OR ("paediatrics"[All Fields] OR "pediatrics"[MeSH Terms] OR "pediatrics"[All Fields]) OR ("pediatrics"[MeSH Terms] OR "pediatrics"[All Fields] OR "pediatric"[All Fields])) AND (("hiv"[MeSH Terms] OR "hiv"[All Fields]) OR ("acquired immunodeficiency syndrome"[MeSH Terms] OR ("acquired"[All Fields] AND "immunodeficiency"[All Fields] AND "syndrome"[All Fields]) OR "acquired immunodeficiency syndrome"[All Fields] OR "aids"[All Fields]) OR ("hiv"[MeSH Terms] OR "hiv"[All Fields] OR ("human"[All Fields] AND "immunodeficiency"[All Fields] AND "virus"[All Fields]) OR "human immunodeficiency virus"[All Fields]) OR ("acquired immunodeficiency syndrome"[MeSH Terms] OR ("acquired"[All Fields] AND "immunodeficiency"[All Fields] AND "syndrome"[All Fields]) OR "acquired immunodeficiency syndrome"[All Fields] OR ("acquired"[All Fields] AND "immune"[All Fields] AND "deficiency"[All Fields] AND "syndrome"[All Fields]) OR "acquired immune deficiency syndrome"[All Fields]))) AND (("rehabilitation"[Subheading] OR "rehabilitation"[All Fields] OR "rehabilitation"[MeSH Terms]) OR ("methods"[MeSH Terms] OR "methods"[All Fields] OR "intervention"[All Fields]) OR ("rehabilitation"[MeSH Terms] OR "rehabilitation"[All Fields] OR "habilitation"[All Fields]) OR ("therapy"[Subheading] OR "therapy"[All Fields] OR "therapeutics"[MeSH Terms] OR "therapeutics"[All Fields]))) AND (impairment[All Fields] OR neurocognitive[All Fields] OR ("Cogn Int Conf Adv Cogn Technol Appl"[Journal] OR "cognitive"[All Fields]) OR sensory[All Fields] OR ("behavior"[MeSH Terms] OR "behavior"[All Fields] OR "behavioural"[All Fields]) OR ("behavior"[MeSH Terms] OR "behavior"[All Fields] OR "behavioral"[All Fields]) OR ("communication"[MeSH Terms] OR "communication"[All Fields]))) AND (("africa south of the sahara"[MeSH Terms] OR ("africa"[All Fields] AND "south"[All Fields] AND "sahara"[All Fields]) OR "africa south of the sahara"[All Fields] OR ("sub"[All Fields] AND "saharan"[All Fields] AND "africa"[All Fields]) OR "sub saharan africa"[All Fields]) OR sub-saharan[All Fields] OR ("africa"[MeSH Terms] OR "africa"[All Fields]))	15 May 2019	PubMed	3431

## Abbreviations

ART: Anti-retroviral therapy
HIV: Human immunodeficiency virus
HIV/AIDS: Human immunodeficiency virus/acquired immune deficiency syndrome
MMAT: Mixed Method Appraisal Tool
PCC: Population Concept Context
UKZN: University of KwaZulu-Natal
